# Fatty acid profile of gamma-irradiated and cooked African oil bean seed (*Pentaclethra macrophylla* Benth)

**DOI:** 10.1002/fsn3.176

**Published:** 2014-10-03

**Authors:** Ifeoluwa Olotu, Victor Enujiugha, Adewale Obadina, Kikelomo Owolabi

**Affiliations:** 1 Department of Food Science and Technology, Federal University of AgricultureP.M.B 2240, Abeokuta, Ogun State, Nigeria; 2 Department of Food Science and Technology, Federal University of TechnologyP.M.B 704, Akure, Ondo State, Nigeria

**Keywords:** Cooking, fatty acid, gamma irradiation, *Pentaclethra macrophylla*

## Abstract

The safety and shelf-life of food products can be, respectively, ensured and extended with important food-processing technologies such as irradiation. The joint effect of cooking and 10 kGy gamma irradiation on the fatty acid composition of the oil of *Pentaclethra macrophylla* Benth was evaluated. Oils from the raw seed, cooked seeds, irradiated seeds (10 kGy), cooked, and irradiated seeds (10 kGy) were extracted and analyzed for their fatty acid content. An omega-6-fatty acid (linoleic acid) was the principal unsaturated fatty acid in the bean seed oil (24.6%). Cooking significantly (*P* < 0.05) increased Erucic acid by 3.3% and Linolenic acid by 23.0%. Combined treatment significantly (*P* < 0.05) increased C18:2, C6:0, C20:2, C18:3, C20:3, C24:0, and C22:6 being linoleic, caproic, eicosadienoic, linolenic, eicosatrienoic, ligoceric, and docosahexaenoic acid, respectively, and this increase made the oil sample to have the highest total fatty acid content (154.9%), unsaturated to saturated fatty acid ratio (109.6), and unsaturated fatty acid content (153.9%). 10 kGy irradiation induces the formation of C20:5 (eicosapentaenoic), while cooking induced the formation of C20:4 (arachidic acid), C22:6 (Heneicosanoic acid), and C22:2 (docosadienoic acid). Combined 10 kGy cooking and irradiation increased the susceptibility of the oil of the African oil bean to rancidity.

## Introduction

African oil bean tree is a member of the family *Leguminosae* and subfamily *Mimosoideae* that grows and occurs naturally in West and Central Africa zones (Enujiugha 2008). This nodule forming tree is commonly found in compound gardens and roadsides as part of agroforestry landscapes, with the seed as the only edible part. The seed has its scientific name as *Pentaclethra macrophylla* and they are important sources of minerals such as sodium, calcium, magnesium, and phosphorus (Enujiugha and Akanbi 2002). They are also rich in calories and protein after parboiling, dehulling, slicing, cooking, soaking, and fermenting for 3–5 days (Enujiugha et al. 2012). The fermented seeds are called “ugba” and are used to prepare different delicacies. African oil bean seed as the name implies contains reasonably high percentage of oil, and the seeds are the source of “owala” butter or “owala” oil used in East Africa for soap, candles, lubricants, and medicinal lotions (Allen and Allen 1981). The oil content of *P. macrophylla* was found to be 52.28% according to Enujiugha and Ayodele-Oni (2003), the composition of different constituents were also reported in percentage by weight as triglycerides (2.1), sterol (23.5), free fatty acids (FFA) (32.9), and monoglycerides (1.6). According to Enujiugha (1990), all these constituents except sterols are hydrolyzed to FFA upon the fermentation of the seed to “ugba.”

Gamma radiation also known as cold pasteurization is a technique of food preservation that has been found to prevent infestation of insects in food products during storage and also their contamination by microorganisms (Farkas 1990). It is also used to inhibit sprouting and delay ripening and senescence. However, some food products need to be irradiated with exceptional requirements such as irradiating them at reduced temperature, in an oxygen-free environment or with combined treatments such as the application of heat and irradiation (Grant and Patterson 1995). The use of joint food processing technologies ensures that food is effectively processed and treatments severity is minimized (Enujiugha et al. 2012). In order to take advantage of the effectiveness of the combination treatment, treatment between 1 and 10 kGy being a form of mild irradiation has been acknowledged to be the best (Campbell-Platt and Grandson 1990). A major processing step in the preparation of ugba; a fermented snack from African oil bean seed is cooking. Cooking and *γ*-irradiation as methods of food preservation are established technologies and their effectiveness could be maximized if both are combined (Olotu et al. 2014). Irradiation effects on food depends on the radiation application dose and dosage which are categorized into low dose (<1 kGy), medium dose (1–10 kGy), and high dose (>10 kGy).

The effect of irradiation on locally stored foods is of utmost importance and an insight into these aspects of storage will help in understanding the shelf life of foods as well as its effects on sensitive nutrients (Bamidele and Akanbi 2013). The absolute link of radiation dosage and resultant changes must be understood as a means toward the comprehensive acceptance of radiated foods (Khattak and Klopfenstein 1989). The stability of food components also needs to be evaluated for the normalization of the process of radiation (Erkan and Ozden 2007). Although irradiation has been found to be a useful process to increase the longevity of several agricultural products, but the effect of gamma irradiation and its combination with cooking on the fatty acids of African oil bean seed has not been investigated. Therefore, this study investigated some changes induced in the fatty acid profile of *P. macrophylla* oil due to gamma irradiation and cooking.

## Materials and Method

### Materials

African oil bean seeds were procured from Imo State, Nigeria. The seeds were inspected visually upon receipt.

### Preparation of samples

The seeds were enclosed in polyethylene bags and irradiated at room temperature to absorb doses of 10 kGy with a gamma irradiation source (cobalt-60) Model GS 1000, Category 4, Panorama Wet Storage Source, Siemen, Germany at SHESTCO, Abuja, Nigeria. The 10 kGy-irradiated sample was divided into two parts and one part was cooked at 100°C for 6 h. A soxhlet apparatus was used to extract oil from the 10 kGy irradiated bean seeds. The solvent used was n-hexane.

### Fatty acid analysis

Fatty acids were determined using gas chromatographic analysis, as described by Enujiugha (1990).

### Esterification with boron trifluoride

The oil extracted from each sample was homogenized by heating gently in a water bath until clear sediment-free oil was obtained. Oil of about 2–3 drops (ca. 250 *μ*g) was put in a screw-capped test tube and 1.2 mL of 0.4 N sodium hydroxide in methanol was added to saponify the oil. The test tube with the contents was heated in a water bath at 60°C for 10 min to dissolve all the fat globules. The mixture was then neutralized with 1.2 mL 0.7 N HCl to release the fatty acids. About 2–3 mL of boron trifluoride (14%) in methanol was added to methylate the fatty acids. Test tube with content was again heated for another 10 min at 60°C. Approximately, 3 mL of petroleum ether (B.P. 40–60°C) was added and the test tube was joggled for about 5 min to effect distinct phase separation. The upper petroleum ether layer was decanted, dried over sodium sulphate (anhydrous), and concentrated. As a control, ˜0.2 g of fatty acid standards was methylated instead of the oil sample.

### Chromatographic analysis

Exactly 1 *μ*L of methylated sample was injected into the gas liquid chromatograph using a microsyringe. The fatty acid methyl esters were analyzed by GLC using Q94 gas chromatograph with JCL 6000 for Windows 2.0 Chromatography Data System (Thermo Fisher Scientific Inc., Waltham, MA). Retention times of separated fatty acid methyl eaters were compared with standards and a plot of the semilog of the value of the relative retention against equivalent chain length was done. The identified fatty acids were quantized by obtaining the product of the response factor and the peak areas. The fatty acids were expressed as the percentage of weight of the total fatty acids.

### Statistical analysis

All procedures were carried out in triplicate and ANOVA was used to analyse the results according to Steel and Torrie (1980). Duncan's multiple range test was used to separate the means and significances were accepted at 5% confidence level (*P* < 0.05). SAS version 9.0 (2008) for windows was used as the statistical software.

## Results

Fatty acid compositions of the oil from the seeds (irradiated, raw, and cooked extracts) are shown in Table1. Linoleic acid (C18:2) was the predominant fatty acid in the oil of the raw seed and the 10 kGy cooked-irradiated seeds. Linoleic acid was absent in the oil of the 10 kGy-irradiated seeds and was 34.71% in the oil of the cooked seed. The low fatty acids in the seeds were with gamilinolenic (C18:3), hemeicosanic (C21:0), eicosadienoic (C20:2), arachidic (C20:4), docosadienoic (C22:2), eicosapentaenoic (EPA) (C20:5), and docosahexaenoic acids (DHA) (C22:6), which were not present in the oil of raw seeds but were present in different levels in the cooked and irradiated samples. The oil extratced from the seeds were subjected to hydrothermal treatment and had the highest linolenic acid (C18:3; 48.40%). EPA (C20:5; 8.49%), linoleic (C18:3; 5.24%) content, and Oleic acid (C 18:1; 8.02%) was the only acid present in the 10 kGy-irradiated sample oil among all these. There was a significant increase in C24:1 (EPA) during 10 kGy gamma irradiation as well as a significant increase in C22:6 (DHA) with the combined treatment. The content of the total unsaturated, polyunsaturated, and total fatty acids of the oil from the raw seed was 94.72%, 78.28%, and 100.28%, respectively, as shown in Table2. The total saturated fatty acid, the monounsaturated fatty acid of the oil from the cooked oil, 10 kGy-irradiated, and cooked and 10 kGy-irradiated African oil bean seed ranged from 0% to 5.35% and 5.24% to 138.7%, respectively.

**Table 1:** Fatty acid profile of the oil of *Pentaclethra macrophylla* expressed as percentage by weight of the total fatty acids

Fatty acid	Raw seed oil	Cooked seed oil	10 kGy-irradiated seed oil	10 kGy-irradiated and cooked oil
Caproic C6:0	0.08^c^ ± 0.01	0.35^b^ ± 0.01		1.26^a^ ± 0.03
Palmitoleic C16:1	2.47^c^ ± 0.17	7.45^a^ ± 0.14		2.93^b^ ± 0.15
Heptaecanic C17:1	0.04^b^ ± 0.01	0.07^a^ ± 0.01		
Stearic CI8:0	0.08 ± 0.00			
Oleic C18:1	8.02^a^ ± 0.03	7.99^a^ ± 0.02	8.02^a^ ± 0.02	
Elaidic C18:1	0.03^b^ ± 0.00	0.25^a^ ± 0.02		
Linoleic C18:2	53.63^b^ ± 1.00	34.71^c^ ± 0.71	78.17^a^ ± 0.07	
Behenic C20:0	0.05^b^ ± 0.01	4.88^a^ ± 0.02		
Linolenic C18:3	24.55^c^ ± 1.09	48.40^a^ ± 0.05	5.24^d^ ± 0.30	31.85^b^ ± 0.20
Lauric C20:1	13.84^a^ ± 0.26			11.87^b^ ± 0.15
Gamlinolenic C18:3		0.17^b^ ± 0.00		8.66^a^ ± 0.30
Hemeicosanic C21:0		0.12 ± 0.03		
Eicosatrienoic C20:3	0.05^c^ ± 0.00	0.07^b^ ± 0.03		0.13^a^ ± 0.00
Eicosadienoic C20:2		0.09^b^ ± 0.00		7.26^a^ ± 0.20
Benenic C22:0	0.04 ± 0.01			
Erucic C22:1	0.06^b^ ± 0.01	3.35^a^ ± 0.09		
Arachidic C20:4		5.62 ± 0.98		
Tricosanicc 23:0	5.06^a^ ± 0.06			
Docosadienoic C22:2		0.26 ± 0.10		
Ligoceric C24:0	0.25^a^ ± 0.17			0.14^a^ ± 0.01
Nervonic C24:1	0.05 ± 0.03			
Eicosapentaenoic C20:5		3.01^b^ ± 0.06	8.49^a^ ± 0.02	
Docosahexaenoic C22:6		3.01^b^ ± 0.00		12.63^a^ ± 0.12

All values are expressed as means of triplicate determinations (mean ± standard deviation). Values along the same row with the same following letters in superscript are not significantly different (*P* < 0.05).

**Table 2:** Summary of fatty acid profile of *Pentaclethra macrophylla* in percentage by weight of the total fatty acids

Fatty acid	RS oil	CS oil	10 kGy oil	10 kGy + CS oil
Total saturated fatty acid	5.56	5.35	0.00	1.40
Monounsaturated fatty acid	16.44	14.13	16.51	14.80
Polyunsaturated fatty acid	78.28	91.79	5.24	138.70
Total unsaturated fatty acid	94.72	110.80	21.75	153.50
UFA/SFA	17.00	20.70	–	109.60
Total fatty acid	100.28	106.39	21.75	154.90

RS oil, oil from raw sample; CS oil, oil from cooked sample; 10 kGy oil, oil from the 10 kGy-irradiated sample; 10 kGy + CS oil, oil from 10 kGy-irradiated and cooked *Pentaclethra macrophylla*.

## Discussion

Oils from vegetables are complex mixtures that contain several compounds and are made up of FFA, triacylglycerols, glycolipids, diacylglycerols, phospholipids, and other minor components (Wu 2007; Adewuyi et al. 2010). Vegetable oil usage is largely centered on the type of fatty acids present in the oil and these fatty acids fall into various lipid categories (Sridhar and Lakshminarayana 1993; Adewuyi et al. 2010). Studies have shown that the African oil bean seed oil is the drying type. Drying oils are triglycerides whose fatty acid constituents are mostly unsaturated bonds (Enujiugha 2003). Linoleic acid was the major fatty acid and the acid with the highest concentration (53.63%) and this result conforms to the studies of Enujiugha (2003) who found out that an omega-6 fatty acid-linoleic acid was the major unsaturated fatty acid in the seed oil (24.55%). Odoemelam (2005) stated that 4–47% of the oil from *P. macrophylla* contained linoleic and oleic acid. It is well known that dietary fat that is abundant in linoleic acid helps to prevent high blood pressure and cardiovascular diseases, such as atherosclerosis and coronary heart disease (Vles and Gottenbos 1989). This shows that the oil of *P. macrophylla* offers potential benefit to health.

The absence of some fatty acids was observed in the raw oil sample but some of these acids were found invariably amongst the three treatments; C18:3 (linolenic acid), C22:6 (docosahexaenoic acid), C21:0 (Heneicosanoic acid), C20:2 (eicosadienoic acid), C20:4, (arachidic acid), C22:2 (docosadienoic acid), C24:1 (nervonic acid), and C18:1 (oleic acid). Gamma irradiation at 10 kGy decreased the linoleic and linolenic acid content of the seed oil, Hau and Liew, (1993) evaluated how irradiation at 10 kGy affected the linolenic and linoleic acid contents of grass prawns. Irradiation at these dosage reduced linoleic acid content by 16%, while its effect on linolenic acid was insignificant. Here *γ*-irradiation caused alterations in the unsaturated and saturated fatty acids of the oil, which showed increase and reduction in the relative amounts of some of the fatty acids present. The present findings agree with previous studies, where it was found that *γ*-irradiation reduced and increased some fatty acids (Afify et al. 2013).

Brewer (2009) reported that the lipids that are affected by irradiation are majorly the two or more double-bonded polyunsaturated fatty acids. Even though polyunsaturated fatty acids have been investigated to have health benefits, a major challenge is their vulnerability to peroxidation (Haghparast et al. 2010). Also in a study by Weber et al. (2007), some fatty acids that were absent in raw fillets were found at reduced levels after heat treatments (C22:0, C22:1, C14:1, and C20:0). Fatty acids react discretely to heat treatments but, in general, saturated fatty acids which are saturated are fairly stable to normal cooking temperatures, but as the unsaturation degree of a fatty acid increases its stability decreases and this makes the most unstable fatty acid to be the polyunsaturated ones (Sioen et al. 2006) and these might be the reason for the variations that were observed in the fatty acid profile of the oil from the cooked sample. Regulska-Ilow and Ilow (2002) stated that thermal treatment such as cooking has been demonstrated to increase the sensitivity of the omega-3-polyunsaturated fatty acid to oxidation Behenic acid was found in trace amounts and this conforms to the study of Achinewhu (1982). The presence of appreciable amounts of behenic and lignoceric acids is not desirable for edible oils (Odunfa 1986). However, Odoemelam (2005) stated that the increase in level of unsaturation makes it worthy for the purpose of cooking and for use as drying oils as a component for the manufacture of varnishes, cosmetics, and paints.

C20:5 (eicosapentaenoic acid) and C22:6 (docosahexaenoic acid) were induced by the cooking of the *P. macrophylla* as they were not present in the raw oil sample. Furthermore, the lipid extraction method of *P. macrophylla* has an effect on the extracted lipids. In a study by Larsen et al. (2010), lipids extracted from cooked fish samples were more than those obtained from the other samples. This might be as a result of bound lipids which were released as free lipids during the cooking process making them unchallenging to extract. The lipids in the raw oil bean might still be bound in the tissue matrix which could have made them hard to extract. The lipid peroxidation results as explained in an earlier research work (Enujiugha et al. 2012) could be the reason for the changes observed in the present study. Many research works have reported the irradiation-induced fatty acid compositional changes (Brito et al. 2002; Yilmaz and Geçgel 2007; Hong et al. 2010). Gamma irradiation at 10 kGy induced the formation of C22:6 (Docosahexaenoic acid) which was also induced by cooking, also this treatment lead to the formation of C18:1 (oleic acid) and significantly reduced C18:3 (linolenic acid) to 5.25% from 24.55%, this could be an indication of the loss of fragments of hydrocarbons and radicals which were produced from trace components and fatty acids upon irradiation(Kim et al. 2004; Al-Masri and Al-Bachir 2007; Hong et al. 2010). Radicals such as thiyl radicals, which are created by ionizing radiation during the repair of radicals, may also interact with unsaturated fatty acids (Geiβler et al. 2003; Hong et al. 2010).

Oleic acid (C18:1) was also found in the 10 kGy-irradiated *P. macrophylla* oil but absent during combined treatment. Hong et al. (2010) reported that the stability of oleic acid in a food matrix upon irradiation at doses ranging from 0 to 60 kGy is as a result of antioxidants such as tocopherol and *β*-carotene in foods which prevents the action of free radicals. This might be the reason why oleic acid was found after irradiation. Docosahexanaenoic acid (C22:6) and eicosapentanoic acid (C20:5) were the most copious omega-3 fatty acids. EPA and docosahexaenoic being polyunsaturated fatty acid are considered to be liable to oxidation in the course of heating and other culinary treatments.

Combined treatment increased C6:0 (caproic acid), C18:3 (linolenic acid), C20:2 (eicosadienoic acid), C20:3 (eicosatrienoic acid), C18:2 (linoleic acid), C24:0 (ligoceric acid), and C22:6 (docosahexanoic acid) and this increase made the oil sample to have the highest total fatty acid content (154.9%) and the highest unsaturated to saturated fatty acid ratio (109.6) as a result of high level of unsaturation, as well as the highest polyunsaturated fatty acid (138.7%). The catalysts of polyunsaturated auto-oxidation are heat, light, trace metals, and enzymes and this process involves the generation of free radicals (Weber et al. 2007). These free radicals increase auto-oxidation by acting with oxygen to form hydroperoxides that breakdown to kindle new free radicals (Weber et al. 2007). The high level of unsaturation in the oil from the combined treated sample indicates the prevalence of unsaturated fatty acid. The oil from the cooked and irradiated *P. macrophylla* congealed at room temperature and unlike others it had a deep yellow color when it was observed as a result of the unsaturation level of the oil (153.5%). Cooking independently increased the unsaturation level in the oil. In general, oils that contain a higher content of unsaturated fatty acids are liable to oxidation than those with lesser amounts (Tan and Che Man 1999), therefore oil obtained from 10 kGy-irradiated and cooked *P. macrophylla* will be less stable and more prone to rancidity than others because it has the highest concentration of unsaturated fatty acid (153.5%). In a previous study (Enujiugha et al. 2012), the FFA of *P. macrophylla* oil sample significantly reduced from 5.22% to 4.43 (% FFA as oleic acid) upon cooking. This might be as a result of the loss of FFA which was volatile in nature leading to decreased FFA, also, the higher FFA in raw samples compared to cooked samples could also be explained by the deactivation of enzymes due to heating which could prevent the liberation of FFA's due to the activity of lipase in cooked samples. In the present study, combining 10 kGy irradiation with hydrothermal treatment increased polyunsaturation and predisposed the oil to oxidative and hydrolytic rancidity.

## Conclusion

Oil of *P. macrophylla* is richer in unsaturated fatty acid than saturated fatty acid. 10 kGy irradiation and cooking made oil extract from African oil bean seed to be more vulnerable to lipid peroxidation (rancidity) because it increased the degree of unsaturation in the fatty acid (more double-bond formation). 10 kGy irradiation, cooking, and combined treatment decreased, prevented, and induced the formation of some of the fatty acids present in the oil, while irradiating African oil bean seeds at 10 kGy led to the loss of 75% of the fatty acid present that were found to be present in the raw African oil bean seed.
